# Detection of Airway Obstruction Caused by Mochi in a Decomposed Body Using Postmortem CT

**DOI:** 10.7759/cureus.71727

**Published:** 2024-10-17

**Authors:** Haruki Fukuda, Takuya Ishikawa, Rie Sano, Yoshihiko Kominato, Hiroyuki Tokue

**Affiliations:** 1 Legal Medicine, Graduate School of Medicine, Gunma University, Maebashi, JPN; 2 Forensic Medicine, Faculty of Life Sciences, Kumamoto University, Kumamoto, JPN; 3 Diagnostic Radiology and Nuclear Medicine, Graduate School of Medicine, Gunma University, Maebashi, JPN

**Keywords:** autopsy, mochi, postmortem computed tomography, rice cake, schizophrenia

## Abstract

Postmortem CT (PMCT) is widely used in forensic investigations to determine the causes of death and is particularly effective in trauma cases and for detecting foreign bodies such as gas or metallic fragments. However, PMCT utility in cases of advanced postmortem changes remains poorly explored. We present the case of a woman in her 60s with a history of schizophrenia who was found in an advanced state of decomposition. PMCT revealed a high-density foreign body in the pharynx, with a CT value of 154.4 HU. During autopsy, a white object, approximately 5 cm in size, was discovered in the pharynx, which was later identified as a mochi, a traditional Japanese rice cake. This led to the conclusion that the cause of death was asphyxiation due to airway obstruction caused by a mochi. This case highlights the diagnostic potential of PMCT in cases of advanced decomposition, particularly for detecting airway obstruction caused by food. Further research and accumulation of such cases are essential to completely evaluate the utility of PMCT in forensic investigations involving advanced decomposition.

## Introduction

Postmortem CT (PMCT) is widely used in forensic investigations to determine the cause of death and is particularly effective in cases involving trauma, as well as in the detection of foreign bodies such as gas, metallic fragments from weapons, or bullets [1-6]. PMCT can provide crucial insights into the cause of death, even in cases in which the body has undergone significant postmortem changes or has been charred. For instance, there have been reports in which PMCT suggested an intraoral gunshot resulting in severe brain damage in charred bodies, revealed subdural hematomas in advanced skeletal remains, and detected cerebral hemorrhage in the left putamen in a body with severe postmortem changes, including advanced putrefaction and autolysis [6-8]. However, the utility of PMCT in cases of advanced postmortem changes has not yet been thoroughly examined. In this report, we present a case of advanced postmortem changes in which PMCT provided crucial insights into the cause of death, specifically airway obstruction caused by food.

## Case presentation

Patient history

The patient was a woman in her 60s who lived alone. She was found in a severely decomposed state in a seated position in the kitchen by a relative who visited her residence. At the time of her discovery, it was autumn, and the recorded room temperature was 30°C. She regularly received assistance from her family with cleaning and meal deliveries. Her last known contact occurred six days prior to her discovery, when a family member visited her home. She had a medical history of schizophrenia, and according to her family, she had experienced past incidents of choking due to rapid eating.

PMCT findings

**Figure 1:**
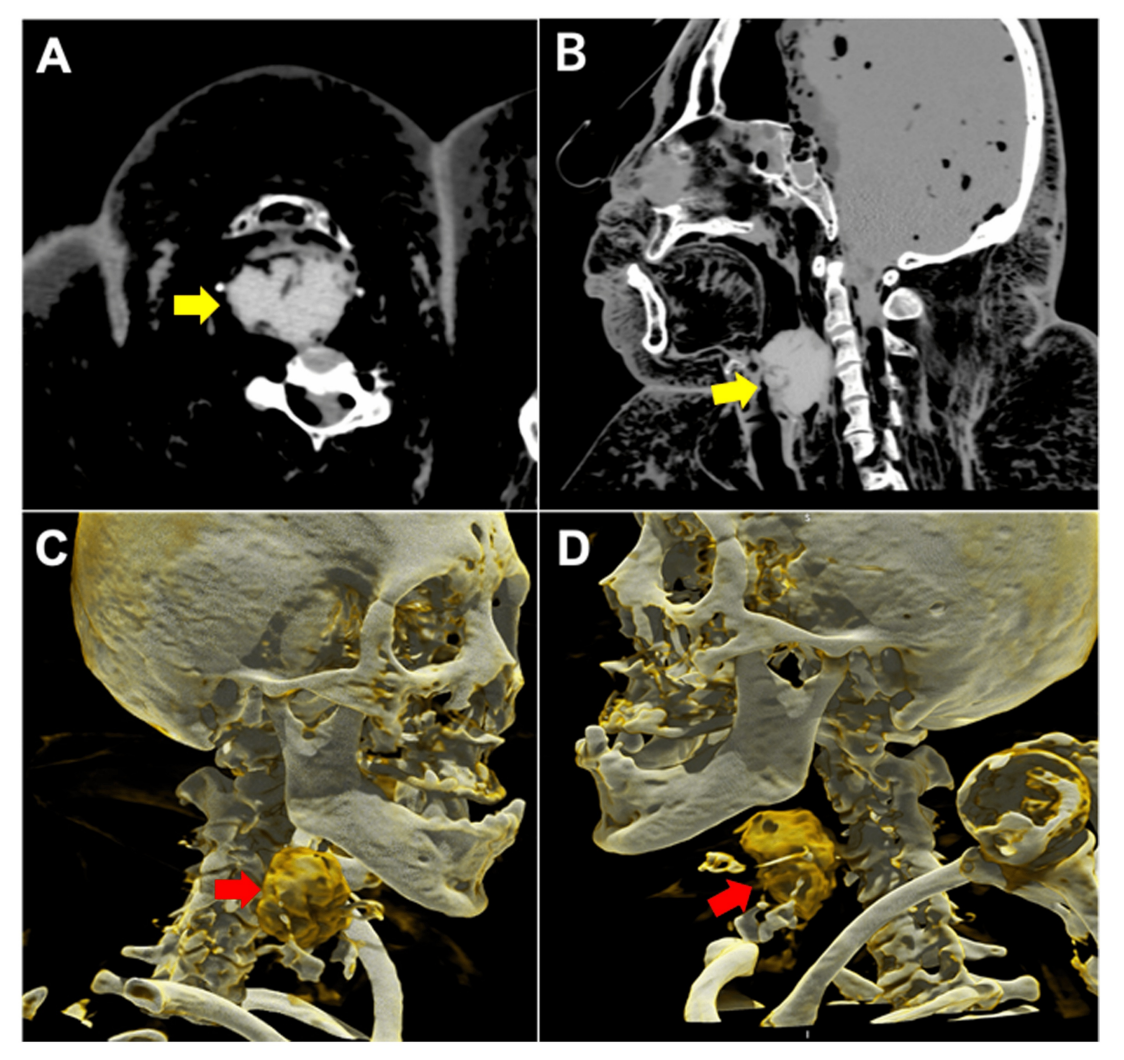
PMCT findings (A, B) Two-dimensional CT images of the head and neck region. (A) Axial view. (B) Sagittal view. A high-density object is observed in the pharynx, indicated by the yellow arrows. Cinematic rendering images of the (C) right lateral view and (D) left lateral view. The foreign body in the pharynx appears yellow-brown, indicated by red arrows. The images were reconstructed using OsiriX MD software. PMCT: Postmortem CT

PMCT scan was performed as a routine procedure prior to autopsy 64 hours after her discovery using a 16-slice CT scanner Alexion/TSX-034A (Toshiba, Tokyo, Japan) with a slice thickness of 0.5 mm and settings of 135 kV, 200 mAs, and 1.5 s/rotation for the head as well as a slice thickness of 1 mm and settings of 135 kV, 150 mAs, and 1.0 s/rotation for the body in the supine position. The PMCT scan revealed a foreign body in the pharynx (Figure 1), with no other significant findings. Using a 3D workstation (Vincent Fujifilm), the volume of the foreign body was measured at 31.8 cm³ (ml), with an average CT value of 154.4 HU.

Autopsy findings

**Figure 2:**
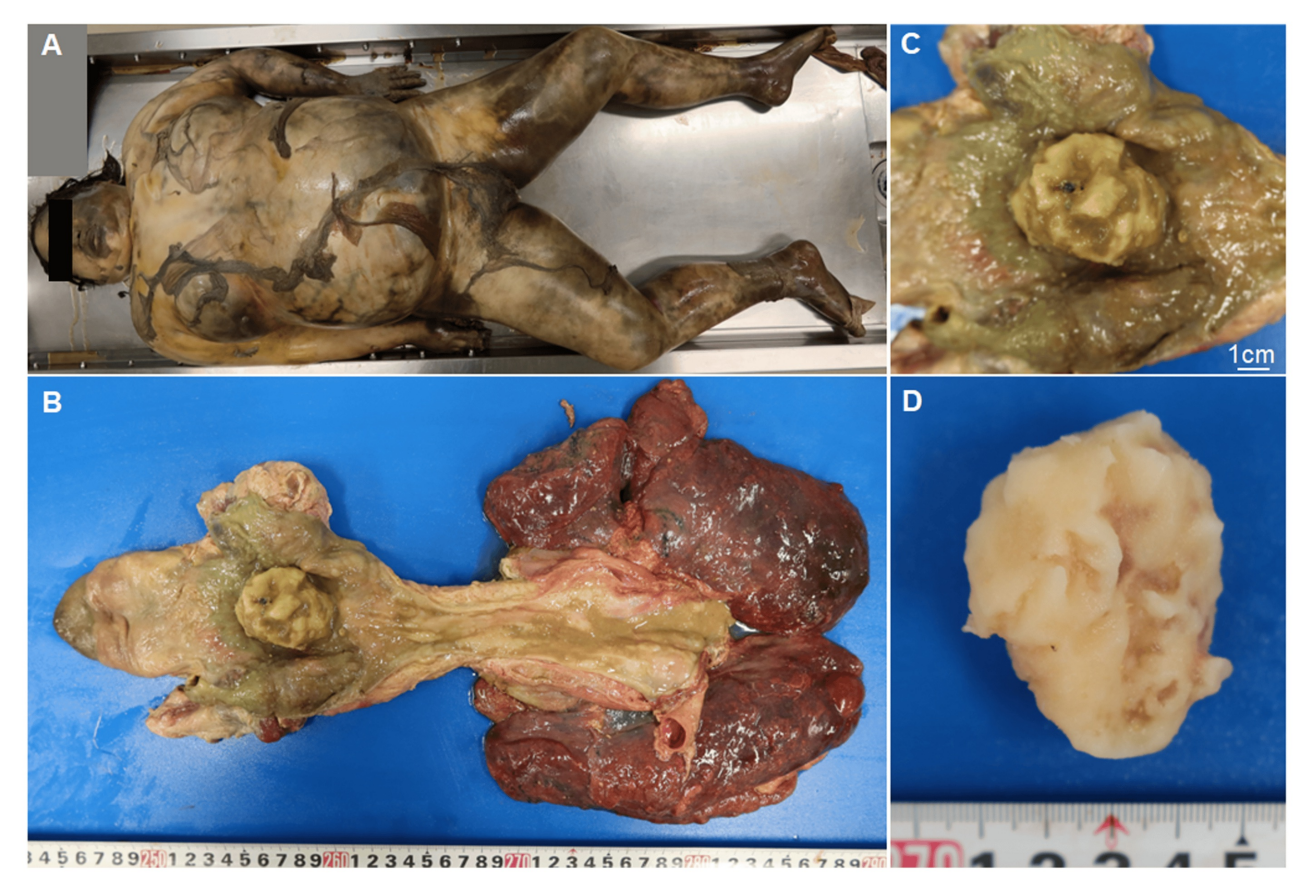
Autopsy findings (A) Anterior view of the external surface of the body; (B) The interior of the pharynx and esophagus showing a 5 cm white object lodged in the pharynx; (C) Close-up of the pharyngeal area from image B; (D) The white object after removal from the body

An autopsy was performed 88 h after the body was discovered. On external examination, the deceased was found to be 157 cm tall and weighed 68.5 kg. The entire body was bloated, and skin slippage was observed in various areas (Figure 2A). Rigor mortis was relaxed in all joints, and no significant external injuries, besides those related to the decomposition, were observed.

Internal examination revealed a white object, approximately 5 cm in size, lodged in the pharynx (Figure 2B, 2C, 2D). Similar substances measuring less than 1 cm and 1-2 cm were found in the esophagus and the stomach, respectively. The cause of death was determined to be asphyxiation owing to airway obstruction. The estimated time from death to autopsy was approximately two weeks. Subsequent investigation revealed a partially eaten mochi within the patient’s residence, suggesting that the white object was likely a piece of mochi. Mochi, a traditional Japanese rice cake, has a sticky and dense texture that can cause choking [9].

## Discussion

In the present case, despite advanced postmortem changes, PMCT imaging raised the suspicion of foreign body aspiration, which was subsequently confirmed to be a mochi through autopsy. Mochi is a traditional Japanese food commonly consumed during the New Year. It is known for its high density, making it a well-recognized cause of choking in the Japanese population [9]. A survey of adult out-of-hospital cardiac arrest cases in Osaka Prefecture from 2005 to 2012 found that 9.5% of cases caused by choking were attributable to mochi [9]. Additionally, 24.5% of out-of-hospital cardiac arrests caused by mochi choking occurred within the first 3 days of the New Year [9]. However, as mochi is frequently consumed outside the New Year’s period, choking incidents, such as in the present case, can occur at any time of the year.

Individuals with schizophrenia are at a higher risk of food-related asphyxiation than those without schizophrenia [10,11]. Dysphagia in schizophrenia generally falls into two categories: changes in eating and swallowing functions due to the disease itself and alterations associated with psychotropic medications. It is common for patients with schizophrenia to eat too quickly or consume large quantities of food inappropriately [10]. In this case, the patient had a history of rapid eating, suggesting that dysphagia related to schizophrenia may have contributed to the choking. It has been reported that 20-25% of all individuals exhibit agonal aspiration of gastric contents, regardless of whether it leads to mortality [12]. Therefore, the mere presence of food in the upper airway during autopsy does not confirm a diagnosis of fatal airway obstruction caused by food. However, in this case, the mochi found in the pharynx was as large as 5 cm, suggesting a high likelihood of airway obstruction due to mochi inhalation. Despite the advanced postmortem changes, mochi was still identifiable in the pharynx, esophagus, and stomach on both CT and during the autopsy. This may be attributed to insufficient chewing as well as the physicochemical properties of mochi, which make it resistant to digestion [13]. Similar cases have been reported where mochi remained undigested in the intestinal tract for over three months, leading to ileus and perforation [14].

Usui et al. reported an autopsy case in which CT imaging showed an impacted mochi in the lower tracheal space, aiding in forensic autopsy planning [15]. However, in the present case, PMCT was useful even in a body with more advanced postmortem changes. The CT density value of mochi has been reported to be approximately 145 HU (range: 120-206 HU), which is consistent with the CT value observed in this case, allowing for easy detection on imaging [13]. Nevertheless, it is challenging to identify the foreign body as mochi based on CT imaging alone. As previously reported, PMCT plays a complementary role in autopsy procedures but is not a complete substitute [2]. Further accumulation of cases is necessary to evaluate the utility of PMCT imaging for bodies with advanced postmortem changes.

## Conclusions

This case highlights the potential of PMCT for detecting airway obstructions such as those caused by mochi, even in bodies with advanced postmortem changes. Thus, PMCT can provide crucial insights into the cause of death, even in cases of significant decomposition. Further research and accumulation of such cases are essential to fully evaluate the utility of PMCT in forensic investigations involving advanced decomposition.
